# Brain Activity-Based Metrics for Assessing Learning States in VR under Stress among Firefighters: An Explorative Machine Learning Approach in Neuroergonomics

**DOI:** 10.3390/brainsci11070885

**Published:** 2021-06-30

**Authors:** Maher Abujelala, Rohith Karthikeyan, Oshin Tyagi, Jing Du, Ranjana K. Mehta

**Affiliations:** 1 Department of Industrial & Systems Engineering, Texas A & M University, College Station, TX 77843, USA; oshin_tyagi@tamu.edu; 2 Department of Mechanical Engineering, Texas A & M University, College Station, TX 77843, USA; rohithkarthikeyan@tamu.edu; 3 Department of Civil and Coastal Engineering, Engineering School of Sustainable Infrastructure and Environment (ESSIE), Herbert Wertheim College of Engineering, University of Florida, Gainesville, FL 32611, USA; eric.du@essie.ufl.edu

**Keywords:** VR, fNIRS, machine learning, firefighters, emergency responders, learning, stress, episodic memory, encoding, retrieval, neuroergonomics

## Abstract

The nature of firefighters’ duties requires them to work for long periods under unfavorable conditions. To perform their jobs effectively, they are required to endure long hours of extensive, stressful training. Creating such training environments is very expensive and it is difficult to guarantee trainees’ safety. In this study, firefighters are trained in a virtual environment that includes virtual perturbations such as fires, alarms, and smoke. The objective of this paper is to use machine learning methods to discern encoding and retrieval states in firefighters during a visuospatial episodic memory task and explore which regions of the brain provide suitable signals to solve this classification problem. Our results show that the Random Forest algorithm could be used to distinguish between information encoding and retrieval using features extracted from fNIRS data. Our algorithm achieved an F-1 score of 0.844 and an accuracy of 79.10% if the training and testing data are obtained at similar environmental conditions. However, the algorithm’s performance dropped to an F-1 score of 0.723 and accuracy of 60.61% when evaluated on data collected under different environmental conditions than the training data. We also found that if the training and evaluation data were recorded under the same environmental conditions, the RPM, LDLPFC, RDLPFC were the most relevant brain regions under non-stressful, stressful, and a mix of stressful and non-stressful conditions, respectively.

## 1. Introduction

Jobs in safety-critical domains, such as firefighting, are often stressful and sometimes life-threatening [1]. Firefighting has been identified as the 5th most dangerous job in the United States [2]. Some of the most prominent occupational stressors faced by firefighters are fear of explosion, exposure to toxic smoke and gases, and fear of making mistakes [3]. This is understandable since their occupation involves high risk, exposes them to life-threatening situations, and the cost of making a mistake could be deadly. Therefore, it is an occupational requirement for firefighters to be at their best even in the most stressful conditions. Firefighters are required to constantly improvise, make quick decisions in a rapidly changing environment, and perform cognitively challenging tasks under immense temporal pressure [4,5]. Therefore, emergency response training is crucial to their ability to perform and survive emergencies. However, the traditional methods of training are limited in immersive experience and repeatability due to cost and safety concerns [6]. Virtual reality (VR)-based training provides an effective solution for this since VR can simulate a variety of emergencies at a relatively low cost [7].

VR-based training allows developing highly adjustable learning systems that are capable of close monitoring of the learners’ cognitive states for effective modulation of training content. Several studies have corroborated the effectiveness of VR in education and training [8,9]. One such study evaluated the use of VR in an episodic memory task where the participant had to encode and retrieve a list of items in a virtual shop [10]. The study found that the VR task resulted in higher levels of presence and motivation within the participants compared to a traditional task, and resulted in improved user performance in VR. In addition to training, VR can also be used for monitoring the specific behavior and cognitive state of individuals. A cross-cultural VR-based investigation of evacuation patterns of crowds in fire emergencies was successfully able to study the influence of the cognitive state of stress on evacuation behavior of individuals [11]. These studies demonstrate the promise of VR to effectively prepare medical professionals for emergencies such as natural disasters, mass casualties, etc. [12,13].

Brain activity and connectivity hold strong potential for monitoring cognitive and physiological states in VR scenarios [14]. Brain activity-based tutoring systems that monitor behavior to assess the cognitive states and workload of the trainee have shown to be very effective for adaptive training [15]. However, very few efforts are being made to integrate neural activity into the VR-based training of firefighters for emergency scenarios. Furthermore, since stress is an important factor in firefighter training, the training system should be able to identify the quality of learning under stress. In classroom settings, memory retrieval under stress is more likely if the encoding of information also occurs in a similar state [16,17]. Therefore, it is important to simulate stress during firefighter training, not only to prepare them for emergencies but to also ensure that memory retrieval is possible in such situations.

Several memory tasks can assess memory encoding and retrieval skills, a subset of which includes episodic memory tasks. Episodic memory is the part of cognition related to remembering time-related past events, and it permits fast information encoding and long-term storage of events [18]. These events could range from being random to personal moments. The encoding, retention, and retrieval of episodic events are associated with brain activity in the temporal lobe, including the hippocampus and surrounding cortical and subcortical structures, and other cortical areas including the prefrontal cortex [18,19,20,21]. To study the association of verbal episodic memory to different brain regions, Shallice et al. [22] isolated brain regions associated with information encoding and retrieving, and reported that information encoding was associated with activity in the left prefrontal cortex and the retrosplenial area, and information retrieval was associated with activity in the right prefrontal cortex and the precuneus. Since brain regions responsible for encoding and retrieving information are different, this allows for the possibility to track the changes in these regions to examine if the person is encoding or retrieving information. However, the surrounding environment or the activity performed could increase and suppress the activation of brain regions associated with encoding and retrieval. For example, acute exercise and stress, which are part of firefighter’s daily work, can affect episodic memory positively or negatively based on when it happens relative to information encoding or consolidation [23]. Acute exercise before encoding, or during early or late memory consolidation enhances episodic memory function but negatively affects episodic memory function if it happens during information encoding. Stress is another factor that affects episodic memory. Firefighters, whose reaction time could decide the fate of the victims, are often required to perform memory retrieval under stressful conditions. Stress increases the activation of several brain regions and impairs the retrieval of episodic memory by interrupting hippocampal-dependent memory processes and cortical function [24,25]. Therefore, it is necessary to investigate how firefighters should be trained to optimize memory retrieval under stress.

Functional brain activity measurement tools, such as electroencephalogram (EEG) and functional near-infrared spectroscopy (fNIRS), can be used to study memory stages and performance. Johannesen et al. [26] found that frontal gamma band during encoding and central theta band during the retention phases were the EEG features most associated with working memory performance that distinguished healthy adults from schizophrenics. They also found that frontal theta band during baseline and frontal alpha band during memory retrieval were main predictors of schizophrenia, with an accuracy of 87%. A study to compare brain function during encoding and retrieval of face-name pairs using fNIRS, reported that brain activity in medial, superior, and middle frontal cortices was significantly higher during retrieval compared to encoding [27]. Similarly, Basso Moro et al. [28] reported a prominent activation increase in the ventrolateral prefrontal cortex during encoding and broader activation in the frontopolar cortex, in addition to the ventrolateral and dorsolateral prefrontal cortex during retrieval, during encoding and retrieval of information in the Logical Memory Test of the Wechsler Memory Scale.

Machine learning (ML) has shown very promising results in many studies investigating data from firefighters or fNIRS tools. For instance, several studies have used ML algorithms, such as decision trees (DT), k-nearest-neighbors (KNN), and support vector machines (SVM), with physiological data such as Heart Rate Variability (HRV), body temperature, and behavior tracking sensors such as accelerometers, to detect mental workload, exertion, and stress in firefighters [29,30,31]. Similarly, other studies have used ML algorithms with other populations to detect task difficulty, mental workload, fatigue, engagement, enjoyment, and user performance [32,33,34,35,36,37,38]. ML algorithms have also been used successfully to classify fNIRS data; for example, they have been used with fNIRS with an average accuracy of 73.79% to recognize positive emotions of participants after watching emotional videos [39]. ML algorithms, such as logistic regression (LR), DT, random forest (RF), SVM, KNN, and multilayer perceptron (MLP), and fNIRS were used to classify whether older adults are just walking or walking while performing a cognitive task with accuracy scores higher than 95% [40]. Additionally, ML techniques, such as SVM, and fNIRS have shown to detect pain with an accuracy of 94.17% [41,42]. The reported algorithmic performances to detect operator states with fNIRS may thus be useful to predict firefighter cognitive states. However, how well ML algorithms assist in detecting where firefighters are in their level of learning, e.g., information encoding vs. information retrieval, using fNIRS in VR has not been previously explored.

With these challenges in mind, this study focused on capturing brain dynamics associated with encoding and retrieval, using fNIRS, while firefighters performed training on a visuospatial episodic memory task within a virtual environment. ML techniques were used to detect if these changes were a response for encoding or retrieving information. To simulate real-life scenarios, one-half of the firefighters performed the memory task under stressful conditions, while the other half performed the task under normal conditions. Neural data, namely temporal hemodynamic features and connectivity metrics, from these two groups, were included to allow the ML algorithms to classify which brain-based metrics could be leveraged to recognize the encoding and retrieval states under either of the environment conditions.

## 2. Materials and Methods

### 2.1. Participants

We recruited 40 firefighters from the local fire station at Bryan, TX. Of those recruited, four were unable to complete the experiment due to VR-sickness, while two others completed an alternate protocol and three participants were excluded from the analysis due to missing data. Therefore, only 31 participants provided useful data pertaining to the current investigation. All participants were healthy males, reflecting the demographics of the fire departments in the region, 30.74±4.19 years old, English-speaking, and 6.9±3.99 years of work experience. All experiment procedures were approved by the Institutional Review Board at Texas A & M University (IRB2019-0943DCR) and proceeded in accordance with the ethics guidelines of the American Psychological Association. Tyagi et. al. details the larger study description and analysis [43]. While the analysis in the larger study focuses on modeling the neural dynamics associated with learning and retrieval under stressful training exercises, this study focuses on detecting encoding and retrieval under normal and stressful environment conditions using machine learning methods.

### 2.2. Protocol

**Figure 1:**
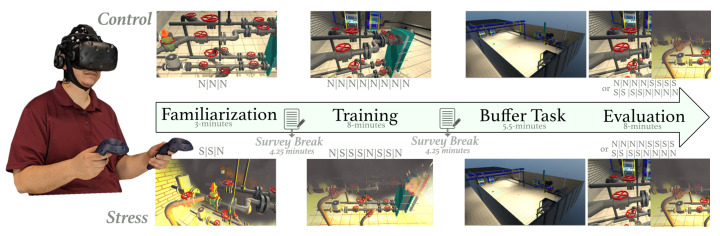
Study Protocol with the familiarization, training, buffer, and evaluation segments. ‘N’ refers to trials completed under normal environmental conditions, and ‘S’ refers to trials completed under stressful environmental conditions. A survey break was used to ask the participants several survey questions.

On informed consent, participants were cast into two training groups, namely control or stress, and led through a VR-based pipe-maintenance task. The virtual interface simulated a chemical power plant with hazards and visual elements that provided participants a salient view of the work environment [44]. The task required participants to execute a sequence of 8 valve operations using the VR hand-held controller. The sequence was shown to the participant before the start of the task. The protocol (see Figure 1) included distinct familiarization, training, buffer, and evaluation segments. During the familiarization period, a virtual cue guided participants through the valve sequence, and participants completed three trials in this phase. All trials were approximately 1-min long. In the training phase, participants were required to repeat the valve sequence under the absence of the virtual cue; however, on committing an error, the interface terminated the experience and the subsequent trial transitioned to familiarization. Participants completed eight trials under this mode, switching to familiarization contingent on their performance errors. Notably, for the stress group, the environmental perturbations (stressors; e.g., fires, alarms, and smoke) were provided most of the time during the familiarization and training period. In the following buffer task, participants were asked to move around the VR environment with no specific objective. The purpose of this segment was to give the participants adequate time to consolidate the memorized sequence [45,46]. In the evaluation period, participants were expected to recall the valve sequence, and execute the entire sequence in VR. Half the trials during this evaluation period were under environmental perturbations while the remainder were under no-stress or control conditions for both training groups. The order of stress and control trials was counterbalanced. Figure 1 has ‘S’ and ‘N’ notes illustrating the environmental condition of the trial, where ‘S’ means that the trial was completed under stressful conditions and ‘N’ means the trial was completed under normal conditions.

### 2.3. Bioinstrumentation

**Figure 2:**
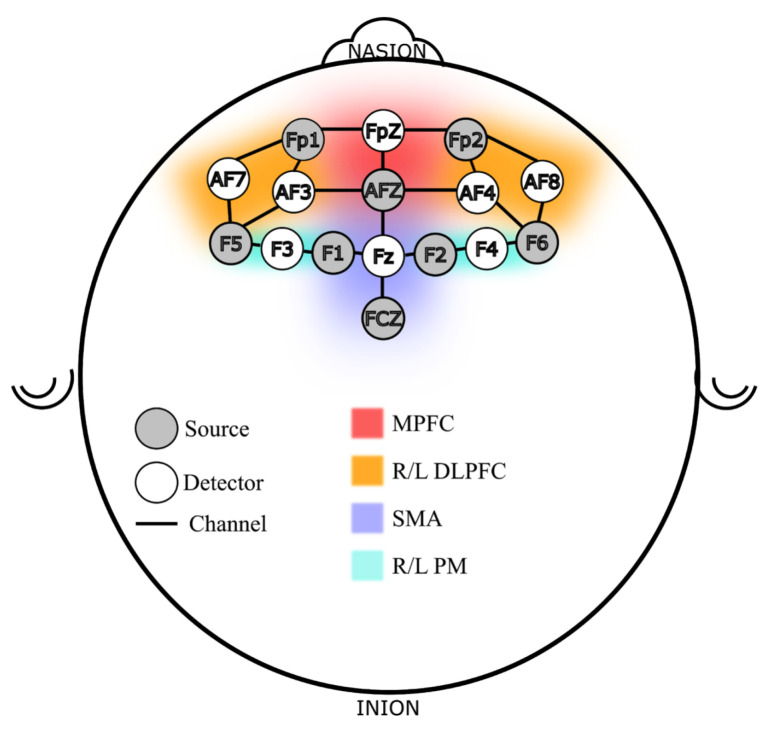
fNIRS Probe Map Design, Channels and Regions of Interest (ROI).

In addition to the VR hand-held controller used to execute the sequence, participants were instrumented with a continuous wave fNIRS device (NIRSport 2, NIRx Medical Technologies, New York, NY, USA) with a probe map focused on cortical locations defined following the 10-10 international systems using a sixteen-probe design. There were 21 channels across a network of brain regions responsible for motor learning and working memory function [47]. Figure 2 shows the probe design used in this study. The probe locations can be roughly divided into six regions of interest (ROI), per Brodmann locations and functions [48,49], three in Brodmann area 9: Medial and Dorsolateral PFC (L/R DLPFC and MPFC), and three in area 6/8: the Premotor (L/R PM) and Supplementary Motor Area (SMA). The DLPFC and the premotor regions were chosen because the PFC works closely with the premotor and supplementary motor areas for complex motor tasks such as sequence learning [50,51,52]. Additionally, stress affects PFC activity and its ability to perform memory related tasks, therefore, PFC is an important region to monitor both for memory and stress related activities [53].

### 2.4. Pre-Processing Brain Hemodynamics

Blood oxygenation changes were captured using the fNIRS device at 8.7 Hz. Near-infrared signals transmitted from 8 emitters were detected by 8 detectors. The transmitted signal characterized hemodynamics across a network of 21 channels. The complete pre-processing workflow was consistent with the steps introduced in [54]. The rawlight intensity ($I_{o}\left(\lambda \right)$) was converted into optical density (OD(λ)) using a log transform [55]. A low-pass filter (3 Hz) was applied to the optical density signal to reduce high-frequency noise. Abrupt peaks or change in the optical density signal were found and corrected using spline interpolation algorithm [56], and smoothed using wavelet transforms [57]. A band-pass filter (0.5−0.016 Hz) was used to reduce the effect of noise and drift. Change in oxygenated, deoxygenated, and total (ΔHbO/R/T) hemoglobin was calculated across all 21 channels using the modified Beer-Lambert principle [58]. The change in concentration of Hemoglobin subtypes was the characteristic signal employed in subsequent machine learning explorations.

### 2.5. Feature Extraction

The pre-processed fNIRS signal was subject to participant-level feature scaling (min-max normalization) before windowing to account for individual differences; and consolidated into sliding windows of duration 15 s, and a step duration of 7.5 s which resulted in 50% overlap between windows. The windows were used to extract relevant time-domain fNIRS features [59]. Additionally, 441 unique temporal features were derived for all 21 channels, and signal types (HbO/R/T). The features include mean, standard deviation, minimum, maximum, kurtosis, skewness, and the area under the curve (AUC) of the temporal brain hemodynamic data. Furthermore, for each window and channel, we derived pairwise Pearson correlation (Corr.) statistics to measure HbO functional connectivity, which resulted in 210 additional connectivity features and increasing the total number of features to 651 features [60]. Table 1 summarizes all the features that were calculated.

### 2.6. Machine Learning Workflow

Machine learning (ML) algorithms were used to detect whether the participant was encoding or retrieving information. The fNIRS features extracted previously were used as the input for the ML algorithms, and the output labels were determined based on the trials. The three familiarization trials were labeled as encoding trials as well as the trials in the training segment in which participants transitioned to familiarization. The eight evaluation trials were labeled as retrieval trials. Each trial was approximately 60 s long; however, the time the participant took to complete the sequence in the segment was variable and limited to a maximum of 60 s. Therefore, the features used in ML were extracted during the first window (15 s overlapping window) to make sure that it represents the participant’s initial state of encoding or retrieving information. This resulted in 422 labeled observations (174 (41.2%) encoding trials and 248 (58.8%) retrieval trials), including 221 (52.4%) no-stress trials and 201 trials (47.6%) stress trials. To examine how the brain regions and ML algorithm’s performance was affected by the activity of the participant (i.e., encoding vs. retrieval) and the environment condition (i.e., stress and no-stress), the encoding and retrieval data were categorized into three groups as shown in Table 2. The first group contains encoding and retrieval data from both conditions (SN—stress and no-stress). On the other hand, the other two groups (N or S) contain data from either the no-stress or the stress condition.

**Figure 3:**
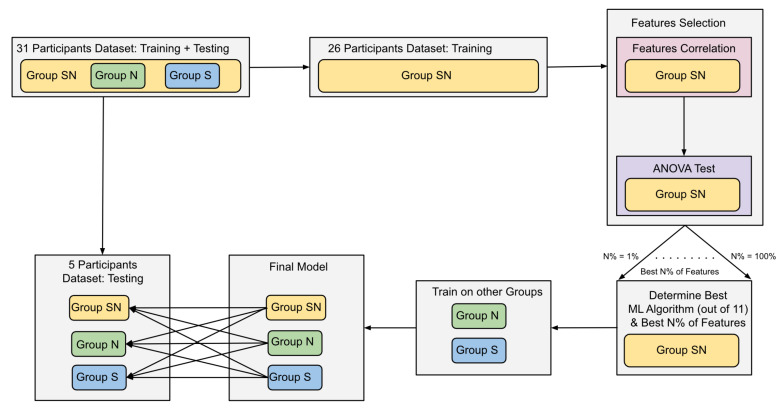
ML workflow diagram. The diagram illustrates the division of the dataset into training and testing. The training data from the Group SN is used to find the best combination of an ML algorithm and N% of the best features to classify encoding and retrieval of information. Once they are determined, they are used to train two models on Group N and Group S data. The final three models are then tested on the testing datasets, and the F1-Score and accuracy of detecting memory encoding and retrieval are reported.

The dataset of 31 participants was divided into training and testing datasets. The training dataset has data of 26 participants and the testing dataset has data of five participants. The training dataset was used to train 11 ML algorithms. The ML algorithms used were Logistic Regression (LR), K-Nearest Neighbors (KNN), Support-Vector Machines (SVM), Gradient Boosting (GB), Extra Trees (ET), Decision Tree (DT), Random Forest (RF), Naive Bayes (NB), AdaBoost (AB), Quadratic Discriminant Analysis (QDA), and Gaussian Process (GP). The parameters of these algorithms were tuned using Randomized Grid-Search over 50-iterations [61,62]. To avoid overfitting the algorithms while tuning the parameters, a 5-folds cross-validation method was used while training the algorithms on the training dataset. To increase the efficiency of the algorithms, two feature selection methods were used. The first method used Pearson’s pairwise correlation to dropout correlated features. The method resulted in no changes as features were not correlated. The second method used the Analysis of Variance (ANOVA) test [62,63]. This test compared every feature individually to the class label, and the importance of the features was evaluated based on the ANOVA F-value. First, the Group SN dataset was used to determine the importance of all features. Then, the algorithms were trained 100 times, each time using the best N% of features, where N is an integer number from 1 to 100. The algorithm and N% of features resulting in the best F-1 score are then trained on Group N and Group S. As shown in Table 2, the encoding and retrieval labels are not balanced, which makes the accuracy score less reliable in interpreting the results. F1-score represents the harmonic mean of precision and recall; thus, it is used to determine the best performing algorithm and features [64]. To examine how the environmental conditions impact brain regions and the performance of machine learning classification. Three models were trained on the training data of the three groups (SN, N, S), and then each model was tested on each of the testing datasets. Based on these final models, the importance of each feature was re-calculated using the permutations of importance measure. This measure iterates through the features and replaces each feature’s values with noise signals and re-calculates the feature’s importance by tracking the changes in the model accuracy. Small to no changes in accuracy indicates the low importance of the feature. Figure 3 illustrates the overview of the machine learning workflow, and the results are discussed in detail in Section 3.

## 3. Results

This section details the results of the ML algorithms in detecting memory encoding and retrieval and highlights the impact of environmental conditions (stress or no-stress) on the performance and transferability of each ML model. We also show in this section which regions of the brain the best ML features come from. To find the best combination of features and model to classify learning, i.e., memory encoding vs. retrieval, each algorithm was trained 100 times with a unique subset of features that were selected using the feature importance ranks provided by the ANOVA tests (see Section 2.6). In each iteration, the Randomized Grid search performed 5-fold cross-validation 50 times to find optimal model parameters; in total we trained and optimized 1100 models. We found that the RF model when using 2% of all available features was the best model in classifying learning based on the resulting F1-score. Table 3 presents the top five models along with their accuracy and F1-scores. The best 2% of the features (i.e., 13 features) were the most common features in those five models and they were found to represent four out of the six ROIs introduced in Section 2.3.

**Figure 4:**
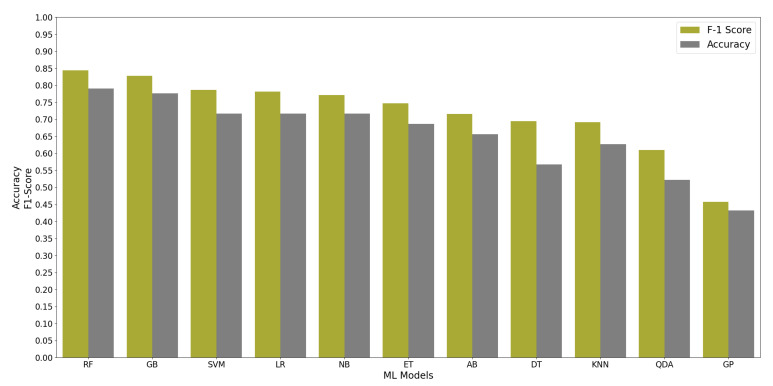
The performance of 11 ML Algorithms when evaluated on Group SN testing dataset using the best 2% of the features determined by the ANOVA test.

**Figure 5:**
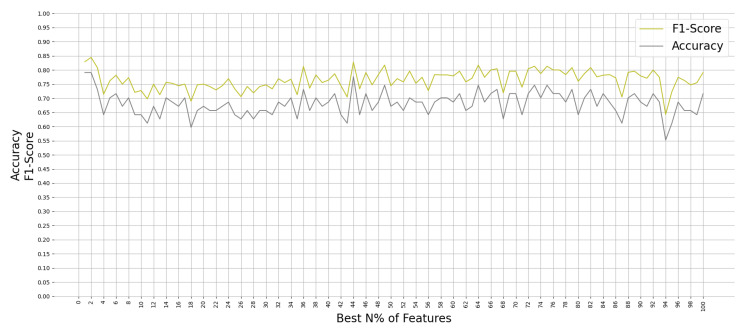
Performance of Random Forest on Group SN testing dataset when trained on N% of the best features.

All ML algorithms performed better than chance in classifying memory encoding and retrieval on the best 2% of the features with accuracy greater than 50% and F-1 scores greater than 0.5, except the Gaussian Process algorithm. Figure 4 shows the test accuracy and F-1 score of all the ML algorithms when used on the best 2% of features. We found that the RF algorithm performed consistently well when relying on different percentages of the input feature set; see Figure 5. Since the RF algorithm and best 2% of the features were the best performing combination, they were trained on Group N and Group S training datasets to classify memory encoding and retrieval as well. Table 4 shows the results of the RF model when trained on one group’s dataset and tested on the others.

We obtained the best results (F-1 score > 0.8) when the training dataset included both Group S and Group N (i.e., Group SN), and when both the training dataset and test dataset included data recorded under similar environmental conditions. For instance, the ML model trained on data collected in non-stressful environment conditions (i.e., Group N) performed poorly (accuracy = 67.65%) when tested on data collected in stressful environment conditions (Group S) compared to data collected in non-stressful (Group N) conditions (78.79%) and both (Group SN) conditions (73.13%). Similarly, the ML model trained on data collected on stressful conditions (Group S) only performed well (accuracy = 76.47%) when tested on data collected under the same condition (Group S).

**Figure 6:**
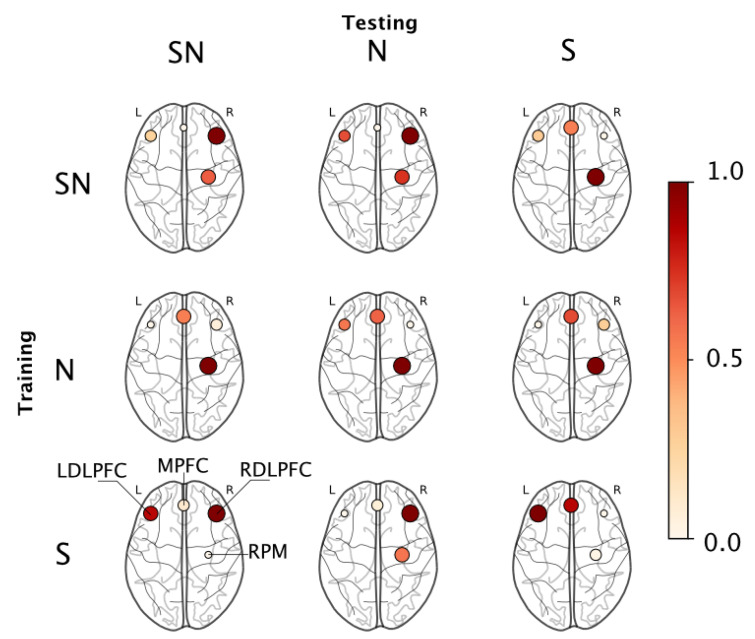
Permutation importance maps for each train-test combination reported in Table 4. The values are normalized with node color and size indicating the relative contribution of each ROI.

To understand what brain regions were responsible for this classification ability, we employed a permutation importance index (see Section 2.6) that ranked features based on their contribution to the resulting model. The ranked features were binned into their corresponding region-of-interest on the basis of the probe map in Figure 2. Each region was assigned a score which is the sum of the importance index of all features derived from that region. Figure 6 shows the regions that were found significant to the classification outcome in each train-test combination using a normalized scale where the marker color and size represent the collective importance of that region. Incidentally, only four regions were found relevant to the outcomes across all permutations, they were—the RDLPFC, MPFC, LDLPFC, and the RPM regions.

## 4. Discussion

### 4.1. General Discussion

In this study, we explored the use of machine learning to discern encoding and retrieval states in firefighters during emergency response training in a VR environment. We operationalized time-series signal metrics and connectivity measures from fNIRS-based brain data towards this state classification problem. Furthermore, we explored the transferability of models built under normal environmental conditions to simulated stress, i.e., off-normal conditions, and vice-versa. We found that our classification method and measures were successful in distinguishing between encoding and retrieval states in our firefighter participant pool with an F1-score of 0.844 and accuracy of 79.10% (see Table 4) when trained and tested on data collected in both stressful and normal conditions (Group SN). We also observed that the model transferred reliably across environment conditions, i.e., stress and no-stress (Group S and Group N), with F1-scores ∈[0.826,0.864] and accuracy scores ∈[76.47%,81.82%]. Among the models trained and evaluated, we found that models that relied on stress data (Group S) did not generalize as well as the rest with F1-scores ∈[0.723,0.764] and accuracy scores ∈[60.61%,68.66%], and models that relied on no-stress data (Group N) did not perform well with stress data, resulting in F1-score of 0.744 and accuracy score of 67.65%. Based on these results, it is recommended that the ML algorithms should be trained on data representing the test scenario.

When we looked at which brain regions were responsible for this classification ability, we found that the RDLPFC region was most important when models were trained and evaluated across all available data (i.e., Group SN), the RPM region was most relevant when models were trained and validated within the no-stress data (i.e., Group N), while the LDLPFC was most important when models were trained and validated within stress data (i.e., Group S); see diagonal elements in Figure 6 and Table 4. On episodic memory tasks such as the one employed in this study, the bilateral prefrontal cortex plays a central role in mediating attentional inhibition and the encoding of a working memory buffer [65]; therefore, we expect activity in these regions to be representative of task-related behaviors. Prior studies that employ fNIRS in investigating cortical networks engaged during episodic working memory tasks similar to our study report the importance of the bilateral dorsolateral prefrontal cortex and ventral PFC regions during encoding and retrieval experiments [27]. On a similar vein, we observed that for most train-test combinations, the PFC regions, including the left and right DLPFC and the medial PFC were significant contributors to the algorithm’s success in distinguishing between learning states.

During the stressor condition, additional cognitive demands are placed on the individual to both filter task-irrelevant information and to stay on task [66]. Previous studies describe the importance of the PFC in these processes (e.g., [67]); therefore, activity across the MPFC, RDLPFC and LDLPFC would also be indicative of the influence of the stressor under these conditions. Several studies have also found that regions peripheral to the RDLPFC, specifically, the right inferior frontal gyrus as a region responsible for attentional inhibition when individuals look to avoid distracting task-irrelevant information (e.g., [68,69]), which further supports this hypothesis. Consequently, we note the importance of these regions especially when validating under the presence of stressor data (e.g., SN-SN, S-S, S-SN, S-N in Figure 6, etc.). On the other hand, under the absence of stress, the RPM played a major role in distinguishing between encoding and retrieval. The primary function of the premotor areas is motor planning and their activation is directly related to the complexity of a given task [51]. The motor areas are also recruited by the PFC for certain tasks requiring complex motor movements or sequence learning [70]. It is possible that while the PFC successfully recruited the RPM for executing the sequence learning task in control condition, this was not the case in the stress condition. Given the constant workload demand of the task, it is possible that the emphasis on the PFC region is limited as the task is mostly routinistic, therefore recruiting only the premotor regions for task-maintenance which could explain how under the N-N condition we find that the RPM was most important to distinguish between encoding and retrieval states.

Our observations point toward unique neural representations during the encoding and retrieval stages of an episodic memory test. We were successful in employing machine learning to capitalize on these differences in distinguishing between the two stages and also to provide some explainability around how those differences changed with environmental conditions. Neural activity has shown strong potential for detecting cognitive and physiological states in VR scenarios [14]; however, there are very few studies that investigate their use in developing adaptive VR training solutions for emergency responders. Our findings signal positively toward the development of VR-based learning platforms that can adapt to the learning state of individual trainees in emergence response (ER) skill development. Such capacity would not only promote personalized learning and adaptation but could one day help accelerate proficiency development across a multitude of ER skills [13].

### 4.2. Limitations

This paper shows a proof of concept that memory encoding and retrieval could be discerned from neuroimaging tools such as fNIRS using machine learning techniques. Our algorithms provide very good accuracy in distinguishing between encoding and retrieval. The labeling mechanism of the fNIRS signals (i.e., encoding or retrieval) was determined based on the requirement of the task. However, memory actions of the participants cannot be controlled, which reduces the confidence in the labels used. For example, the participant is asked to retrieve the sequence but they might be distracted and not start retrieving the sequence until a few seconds later. Additionally, it should be noted that the study was conducted in VR with a small population in a lab environment. The firefighter participant pool was not diverse, and all of them were male participants in the same age range. One expected downside is that the ML algorithm might not perform as well if it is tested on participants of different ages and or sex [71]. Additionally, fNIRS is an intrusive device that is uncomfortable to wear for long hours, and it would be impractical to use in real-life emergency training scenarios in its current form. This could limit the transferability of our methods to a more diverse population in real-life scenarios. To use our methods in adaptive training, our methods need to work in real time in order to provide the input needed by the adaptive system to make the decision on how to change the training scenario. Currently, our ML approach in this study was designed to work offline after manually engineering the features, so it needs to be changed to provide real-time classification. Nevertheless, our algorithm reliably predicts memory encoding and retrieval, and it indicates that our study is a step in the right direction towards building an adaptive training solution.

## 5. Conclusions

Firefighters work under dangerous, stressful conditions that require them to stay attentive and make quick decisions in rapidly changing environments. The training they receive is crucial to their ability to perform and survive in these environments. VR-based training systems have proven to be effective in several fields. However, there are not many studies that explore the use of VR-based training in emergency scenarios and explore the use of neural activities to monitor user’s learning states. This paper investigated the use of machine learning methods to classify memory encoding and retrieval states in firefighters during a pipe-maintenance task and illustrated which regions of the brain were responsible for the performance of the ML method. It also explored the impact of the environmental conditions (i.e., stress and no-stress) of the training data on the generalizability of ML algorithm when tested on new data that was recorded in a similar or a different environmental condition. We were able to achieve an F-1 score of 0.844 and an accuracy of 79.10% when the ML algorithm was trained and tested on data collected in diverse environmental conditions. With consideration to the indicated limitations of the study, our ML techniques could take us a step further towards the goal of developing an adaptive VR-based learning platform that can adapt to the learning state of emergency response trainees.

## Figures and Tables

**Table 1:** Features extracted from brain hemodynamics data.

Metric	Equation
Mean (μ)	$\frac{1}{n}\sum_{i=1}^{n}x_{i}$
Variance ($\sigma^{2}$)	$\frac{\sum_{i=1}^{n}{\left(x_{i}-\mu \right)}^{2}}{n}$
Maximum	max(x)
Minimum	min(x)
Kurtosis	$\frac{\sum_{i=1}^{n}{\left(x_{i}-\mu \right)}^{4}/n}{{\left[\sum_{i=1}^{n}{\left(x_{i}-\mu \right)}^{2}/n\right]}^{2}}$
Skewness	$\frac{\sum_{i=1}^{n}{\left(x_{i}-\mu \right)}^{3}/n}{{\left[\sum_{i=1}^{n}{\left(x_{i}-\mu \right)}^{2}/n\right]}^{1.5}}$
AUC	∫xdt
Corr.	$\frac{\sum_{i=1}^{n}\left(x_{i}-\mu_{x}\right)\left(y_{i}-\mu_{y}\right)}{\sqrt{\sum_{i=1}^{n}{\left(x_{i}-\mu_{x}\right)}^{2}}\sqrt{\sum_{i=1}^{n}{\left(y_{i}-\mu_{y}\right)}^{2}}}$

**Table 2:** Encoding and Retrieval Data Groups for ML classification. SN refers to data collected during both stress and no-stress conditions. Similarly, N refers to no-stress condition data, and S refers to stress condition data.

Groups	Encoding under No-Stress	Retrieval under No-Stress	Encoding under Stress	Retrieval under Stress
SN	97	124	77	124
N	97	124	0	0
S	0	0	77	124

**Table 3:** The top five models on the testing dataset of Group SN in classifying memory encoding and retrieval. The models are ranked based on their F-1 score on the testing dataset.

Model	Percentage of Best Features	Accuracy	F-1 Score	Precision	Recall
RF	2%	79.10%	0.844	0.760	0.950
ET	88%	76.12%	0.830	0.722	0.975
RF	1%	79.10%	0.829	0.810	0.850
GB	2%	77.61%	0.828	0.766	0.900
RF	44%	77.61%	0.828	0.766	0.900

**Table 4:** Accuracy and F-1 scores of the Random Forest algorithm (when using best 2% of the features) after training it on one group and testing on others.

Training Group		Testing Group		
		**SN**	**N**	**S**
	**SN**	79.10% F-1 = 0.844	81.82% F-1 = 0.864	76.47% F-1 = 0.826
	**N**	73.13% F-1 = 0.786	78.79% F-1 = 0.829	67.65% F-1 = 0.744
	**S**	68.66% F-1 = 0.764	60.61% F-1 = 0.723	76.47% F-1 = 0.810

## Data Availability

The dataset presented in this study is available for qualified researchers upon reasonable request from the corresponding authors.
